# Effect of clinical status on survival in patients with borderline or locally advanced pancreatic adenocarcinoma

**DOI:** 10.1186/s12957-019-1637-1

**Published:** 2019-06-04

**Authors:** Pauline Duconseil, Jonathan Garnier, Victoria Weets, Jacques Ewald, Ugo Marchese, Marine Gilabert, Laurence Moureau-Zabotto, Flora Poizat, Marc Giovannini, Jean-Robert Delpero, Olivier Turrini

**Affiliations:** 1 Department of Surgical Oncology, Institut Paoli Calmettes, Aix-Marseille University, CRCM, 232 Boulevard de Sainte Marguerite, Marseille, 13009 France; 2 Department of Oncology, Institut Paoli-Calmettes, Marseille, France; 3 Department of Radiotherapy, Institut Paoli-Calmettes, Marseille, France; 4 Department of Pathology, Institut Paoli-Calmettes, Marseille, France; 5 Department of Endoscopy, Institut Paoli-Calmettes, Marseille, France

## Abstract

**Objective:**

To determine the effect of clinical status (weight variation and performance status [PS]) at diagnosis and during induction treatment on resectability and overall survival (OS) rates in patients with borderline resectable (BRPC) or locally advanced pancreatic cancer (LAPC).

**Methods:**

From 2005 to 2017, 454 consecutive patients were diagnosed with LAPC or BRPC. We evaluated the PS (0–1 or 2–3), body mass index at diagnosis, and weight loss (WL) > 5% at initial staging and after induction treatment and separated continuous weight loss (CWL) from weight stabilization.

**Results:**

A total of 294 patients (64.8%) presented with WL, and 57 patients (12.6%) presented with a PS of 2–3. At restaging, 60 patients (13.2%) presented with CWL. Independent factors that poorly influenced the OS were a PS of 2–3 at diagnosis (*P* < .01), CWL at restaging (*P* < .01), and absence of resection (*P < .*01). Factors independently impeding resection were LAPC (*P* < .01), PS > 1 at diagnosis (*P* < .01), and CWL (*P* = .01). In total, 142 patients (31.3%) underwent pancreatectomy. Independent factors that poorly influenced the OS in the resected group were PS > 0 at diagnosis (*P* = .01) and obesity (*P* < .01). For the 312 unresected cancer patients (68.7%), CWL (*P* < .01) was identified as an independent factor that poorly influenced the OS.

**Conclusion:**

Clinical parameters that are easy to measure and monitor are independent factors of poor prognosis. The variation of weight during the induction treatment, more than WL at diagnosis, significantly precluded resection and was an independent factor of shorter OS in unresected patients.

## Introduction

Weight loss (WL) is a cardinal sign of cancer, especially in digestive malignancies. Cachexia is commonly defined by WL > 5% within 6 months, which is sufficient enough to alter immunologic, cardiac, and respiratory functions [1]. Weight loss is observed in 50 to 80% of patients with pancreatic ductal adenocarcinoma (PDAC) [2–4], which is projected to become the second cause of cancer deaths within the next 10 years. Decreased oral intake, induced catabolism by the disease [5, 6], depression felt among the patients but also exocrine and endocrine insufficiencies are considered to be the causes of malabsorption and diabetes imbalance [7]. The biological mechanisms of the effect of WL on outcome and prognosis have not been elucidated yet [8], but have partially been explained by factors, such as the lesser doses of chemotherapy, the lesser duration of treatment, and the more side effects that these weakened patients experienced [9]. Moreover, skeletal muscle loss and adipose wasting impair functional recovery and healing and could increase postoperative complications such as postoperative pancreatic fistula, therefore prolonging hospital stay and affecting oncological outcomes [10–13]. The negative effect of WL has also been correlated with the decrease of the performance status (PS) and with quality of life [1, 14]. Locally advanced pancreatic cancer (LAPC) and borderline resectable pancreatic cancer (BRPC) are two entities of PDAC for which clinical presentation, therapeutic strategy, and prognosis are in between upfront resectable PDAC and metastatic disease. Patients usually undergo a medical treatment, for example, chemo- and/or radiotherapy, as an induction treatment and are then reevaluated by a multidisciplinary staff before being referred either to the surgeon with an “intention to resect” or to the oncologist to continue undergoing medical treatment. This long therapeutic sequence together with the tumor biology itself contributes to patients’ clinical status modifications that could strongly affect the therapeutic strategy and consequently the overall survival (OS). To date, we do not have any strong and reliable clinical or biological tool to predict which patients will benefit from his induction treatment and be selected for surgery at re-staging. As an example, weight gain during preoperative treatment has been highlighted as a strong predictor of eventual surgery [15], and we hypothesized that continuous weight loss (CWL) during the induction treatment was a sign of poor prognosis. Thus, we sought to determine the effect of clinical status (WL and PS [16]) at diagnosis and during induction treatment on resectability and OS in patients with BRPC or LAPC.

## Methods

### Patient selection

From January 1, 2005, to December 31, 2017, 454 consecutive patients were diagnosed as having LAPC or BRPC (according to the 2017 National Comprehensive Cancer Network Guidelines [17]) at Institut Paoli-Calmettes (Marseille, France) and consequently underwent an induction treatment. All patients’ data were entered prospectively into a clinical database approved by our Institutional Review Board. Patients eligible for the present study had a histologically proven PDAC and did not undergo up-front pancreatectomy. All patients were initially staged by physical examination (including PS and weight) and underwent thoracoabdominal computed tomography scanning (CT scan), and patients’ CA 19-9 serum levels were also measured. Due to the period of inclusion, neither liver magnetic resonance imaging nor positron emission tomography scan was routinely performed. All patients underwent an induction treatment (i.e., chemotherapy or chemoradiation) according to our period of inclusion and by multidisciplinary staff decision; endoscopy with ultrasound sonography and fine needle aspiration was performed to obtain histological confirmation prior to undergoing medical treatment.

### Restaging, surgery, and adjuvant treatment

After induction treatment, patients were restaged clinically (visit with the oncologist, including the measure of weight) and by getting a new thoracoabdominal CT scan, and the multidisciplinary staff made the final decision either to continue medical treatment or to perform an explorative laparotomy with curative resection intent. Indeed, surgery exploration was decided if no disease progression had been diagnosed on the CT scan. If there was a radiological doubt on liver metastases, a hepatic MRI could be performed, but it was not done routinely in the restaging sequence. In case of surgery, a thorough abdominal exploration was first performed to rule out carcinomatosis, liver metastasis, and, more recently, para-aortic lymph node metastasis [18], precluding pancreatectomy. If appropriate, pancreatectomy with en bloc venous resection was performed as already described [19]. All specimens were inked to facilitate margin assessment. According to postoperative courses and patients’ clinical status, an adjuvant gemcitabine-based chemotherapy was performed for 6 months.

### Study parameters

The variables evaluated included the following: age; gender; PS (0–1 or 2–3); body mass index (BMI) at diagnosis defining underweight (< 18.5 kg/m^2^), normal weight (18.5–24.9 kg/m^2^), overweight (25–29.9 kg/m^2^), and obesity ( ≥30 kg/m^2^); WL at diagnosis defined by a weight loss > 5% (when compared with the usual weight within the last 6 months prior to diagnosis or first disease symptoms); variation of WL between diagnosis and restaging (stabilization or increase in weight were thus opposed to CWL, defined as a decrease in weight between initial and restaging evaluation, in patients already diagnosed with WL at diagnosis), tumor location (i.e., head, body, tail); abdominal pain; jaundice; biliary stenting; type of induction treatment (i.e., chemotherapy or chemoradiation); and CA 19-9 serum level (after jaundice resolution, before induction treatment, and before surgery). In case of pancreatectomy, type of surgery (i.e., pancreaticoduodenectomy, total pancreatectomy, or distal pancreatectomy), vascular resection (venous and/or arterial), margin status (a resection margin inferior to 1.5 mm was considered as involved margin (R1) [20], lymph node status (i.e., positive or negative nodes), perineural invasion, disease staging established according to the TNM 8th classification of the American Joint Committee on Cancer [21], overall morbidity according to Clavien-Dindo Classification [22], mortality (30 and 90 days after surgery or before hospital discharge), length of hospital stay, readmission, and administered adjuvant treatment were also recorded.

### Statistical analysis

Data analyses were performed using GraphPad Prism software version 5.0d (GraphPad Software Inc., La Jolla, CA) and SAS statistical software version 9.1 (SAS Institute, Inc., Cary, NC). The categorical factors were compared using Fisher’s exact test; the continuous variables were compared using the Student’s *t* test. The association of categorical factors with OS was assessed using the Kaplan-Meier method (based on the date of diagnosis and the date of death or status at the censored date, November 1, 2018) and was tested using the Wilcoxon test. Statistical significance was set at *P* value < .05. Prognostic factors with *P* < .1 in a univariate analysis or known to affect PDAC survival were included in a multivariable regression model to determine the independent factors.

## Results

### Entire cohort

**Fig. 1:**
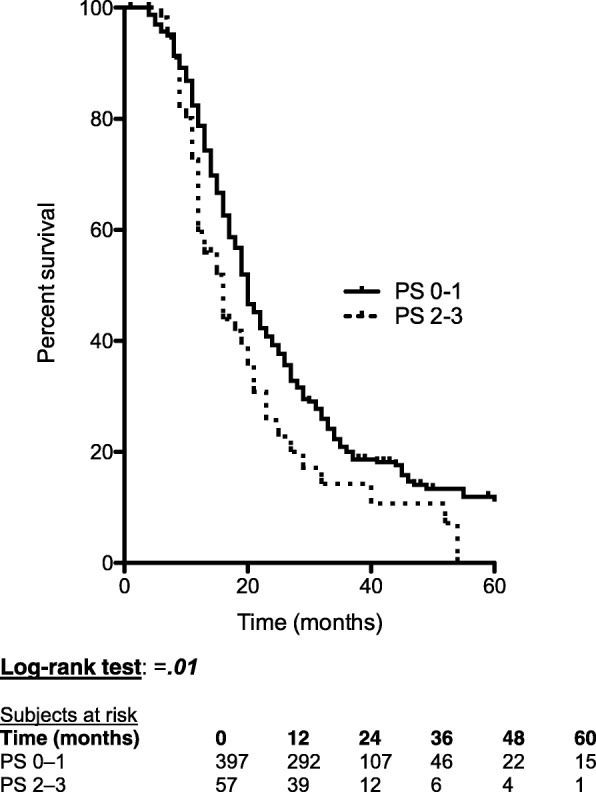
Effect of performance status at diagnosis on overall survival

**Fig. 2:**
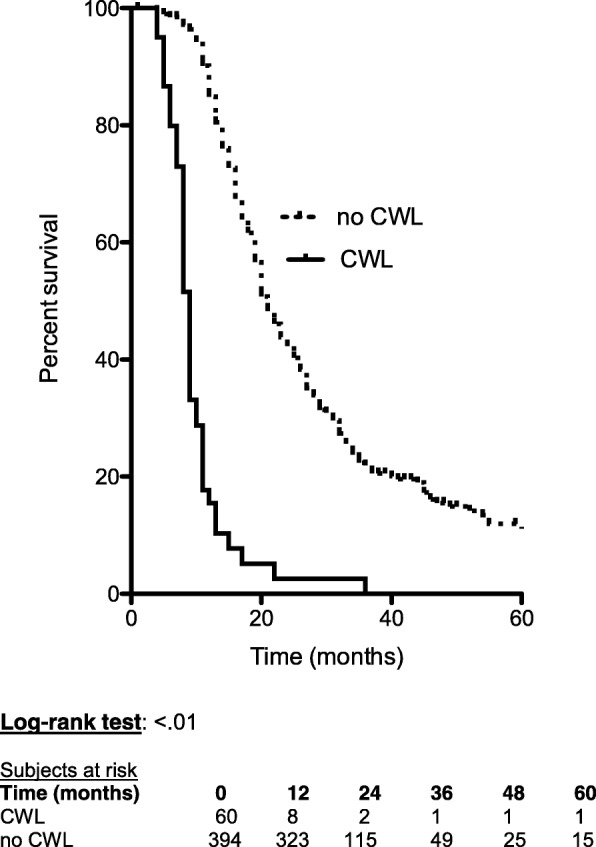
Effect of continuous weight loss (CWL) at restaging on overall survival when comparing with stable or regaining weight (no continuous weight loss, no CWL)

All the patients included in this retrospective study had received an induction treatment, which could be (a) chemo-radiation in 79 patients (17.4%) based on intensity-modulated fractionated radiotherapy combined with concurrent chemotherapy, with capecitabine (800 mg/m^2^ twice daily, 5 days/week). The total dose of radiotherapy was 45 Gy in 25 fractions/5 weeks for patients with BRPC, and this dose was increased to 54 Gy in 30 fractions/6 weeks for LAPC; (b) chemotherapy alone in 375 patients (82.6%), based on Folfirinox in 258 patients (56.8%), Gemcitabine in 89 patients (19.6%), and other regimen in 28 patients (6.2%). The mean follow-up was 22 months. The median survival time of the 454 patients was 20 months. The OS at 1, 3, and 5 years were 76%, 19%, and 10%, respectively. According to the initial staging, WL was observed in 294 patients (64.8%), and a PS of 2–3 was noted in 57 patients (12.6%). At restaging, 60 patients (13.2%) had lost weight during the induction treatment and where thus tagged as with CWL (Table 1). In a multivariate analysis (Table 2), independent factors that poorly influenced the OS were as follows: a PS of 2–3 at diagnosis (*P* < .01) (Fig. 1), CWL at restaging (*P* < .01) (Fig. 2), and absence of resection (*P < .*01); while they were identified as significant factors in a univariate analysis, underweight and WL were not considered significant factors in a multivariate analysis.

**Table 1:** Clinical characteristics of the 454 patients

Sex ratio, female/male	0.96
Mean age (± SD)	65 (± 10)
Mean BMI (± SD)	23.6 (± 4.16)
< 18.5 (%)	37 (8.2)
18.5–24.9 (%)	282 (62.1)
25–29.9 (%)	105 (23.1)
≥ 30 (%)	30 (6.6)
Performance status at diagnosis	
0–1 (%)	397 (87.4)
2–3 (%)	58 (12.6)
Weight loss	
Weight loss at diagnosis (%)	294 (64.8)
Continuous weight loss at restaging (%)	60 (13.2)
Jaundice (%)	264 (58.1)
Biliary stenting (%)	272 (59.9)
Abdominal pain (%)	293 (64.5)
Mean CA 19-9 serum level (UI)(±SD)	
At diagnosis (after jaundice resolution)	1648 (± 6020)
Tumor location	
Head (%)	315 (69.4)
Body (%)	102 (22.5)
Tail (%)	37 (8.1)
Tumor staging	
Borderline	336 (74)
Locally advanced	118 (26)
Induction treatment (%)	454 (100)
Chemotherapy (%)	375 (82.6)
Chemoradiation (%)	79 (17.4)
Resection (%)	142 (31.3)

*BMI* body mass index, *CA 19-9* carbohydrate antigene 19-9

**Table 2:** Entire cohort: univariate and multivariate analysis of factors influencing overall survival (*n* = 454)

	Univariate	Multivariate	
	*P*	Hazard ratio	*P*
Underweight (BMI < 18.5 kg/m^2^)	.37	–	
Performance status (0–1 versus 2–3)	*< .01*	1.6 (1.21–2.11)	*< .01*
Weight loss at diagnosis	*< .01*	–	
Continuous weight loss at restaging	*.01*	9.56 (6.32–14.5)	*< .01*
Jaundice	.28	–	
Pain	.12	–	
CA 19-9 level	.08	–	
Tumor location (head versus body/tail)	.54	–	
Tumor staging (BRPC versus LAPC)	*< .01*	–	
Resection	*< .01*	.38 (.284–.513)	*< .01*

Entries in italic are significant, even if only in univariate analysis

*BMI* body mass index, *CA 19-9* carbohydrate antigene 19-9, *BRPC* borderline resectable pancreatic cancer, *LAPC* locally advanced pancreatic cancer

At restaging, 204 patients were considered as eligible to surgery and underwent laparotomy. Sixty-two could not be resected because of local extension (25 patients; 40.3%), liver metastases (13 patients; 21%), carcinomatosis (13 patients; 21%), or para-aortic lymph node metastases (11 patients; 17.7%).

### Resected patients

Among the 204 patients for whom resection had been decided by the multidisciplinary staff, 142 patients (69.6%) underwent pancreatectomy after a 12-week (range, 7–44) median delay from the initiation of induction treatment. In a multivariate analysis, factors that independently impeded resection were the following: LAPC (*P* < .01), PS > 1 at diagnosis (*P* < .01), and CWL(*P* = .01). Only 2 patients (1.4%) with a PS of 2 at diagnosis underwent resection, whereas none of the patients with a PS of 3 at diagnosis. Seventy-four patients (52%) underwent a complete sequence of adjuvant chemotherapy (Table 3). During the follow-up period, 75 patients (52.8%) died from PDAC recurrence. The median survival time of the 142 patients was 29 months. The OS at 1, 3, and 5 years were 86%, 43%, and 31%, respectively. In a multivariate analysis (Table 4), independent factors that poorly influenced the OS were the following: PS > 0 at diagnosis (*P* = .01), obesity (*P* < .01), R1 resection (*P* = .04), lymph node invasion (*P* = .04), and absence of adjuvant treatment (*P < .*01).

**Table 3:** Resected patients: surgery, postoperative courses, and pathological findings (*n* = 142)

Type of surgery	
PD	102 (71.8)
Total pancreatectomy	6 (4.3)
Distal pancreatectomy	34 (23.9)
Vascular resection (%)	95 (66.9)
Venous resection	79
Arterial resection	5
Venous + arterial resection	11
Morbidity (%)	98 (69)
Grade B or C POPF	17 (12)
Hemorrhage	12 (6.4)
Reintervention	13 (9.2)
Mortality	
30 days (%)	9 (6.3)
90 days (%)	12 (8.5)
Length of hospital stay (days) (± SD)	16 (± 7.71)
Readmission (%)	15 (10.6)
T3/T4 stage	100 (70.4)
Mean number of examined lymph nodes (± SD)	15 (± .74)
Lymph node invasion (%)	86 (60.6)
R1 status (%)	37 (26)
Adjuvant treatment (%)	74 (52.1)

*PD* pancreaticoduodenectomy, *POPF* postoperative pancreatic fistula

**Table 4:** Resected patients: univariate and multivariate analysis of factors influencing overall survival (*n* = 142)

	Univariate	Multivariate	
	*P*	Hazard ratio	*P*
Underweight (BMI < 18.5 kg/m^2^)	.44	–	
Obesity (BMI ≥ 30 kg/m^2^)	*.01*	2.99 (1.34–6.66)	*<.01*
Performance status (1 versus 0)	*< .01*	1.91 (1.15, 3.17)	*.01*
Weight loss at diagnosis	.14	–	
Continuous weight loss at restaging	.91	–	
Jaundice	.87	–	
Abdominal pain	.68	–	
CA 19-9 level	.34	–	
Tumor location (head versus body/tail)	.86	–	
Tumor staging (BL versus LA)	*< .01*	–	
R1 status		1.75 (1.03–2.96)	*.04*
Lymph node invasion		1.66 (1.01–2.74)	*.04*
Adjuvant treatment	*< .01*	.5 (.299–.843)	*< .01*

Entries in italic are significant, even if only in univariate analysis

*BMI* body mass index, *CA 19-9* carbohydrate antigene 19-9, *BL* borderline, *LA* locally advanced

### Unresected patients

For the 312 unresected patients (68.7%), the median survival time was 17 months. The OS at 1, 3, and 5 years were 71%, 8%, and 1%, respectively. In a multivariate analysis, CWL (*P* < .01) was identified as an independent factor that poorly influenced the OS.

## Discussion

### Study highlights

In this selected population of patients with BRPC and LAPC, we found that clinical parameters that are easy to measure and monitor were independent factors of poor prognosis. Indeed, the CWL during the 3-month induction treatment period, more than WL at diagnosis, significantly precluded resection and was an independent factor of shorter OS in unresected patients. PS was an independent factor of poor prognosis in all patients, with different thresholds according to the population: the fittest patients (PS < 1) were more eligible to resection and had a better OS, whereas the more fragile patients (PS 2–3) had worse OS rates in the entire cohort analysis. PS and WL are related [23] and are routinely monitored, but are rarely used to screen the patients and indicate enhancement programs. Thus, programs of physical activity during neoadjuvant treatment have been evaluated by Parker et al. [24] in pancreatic cancer patients, as an extent to a modern enhanced recovery after surgery concept. This study revealed that patients undergoing neoadjuvant treatment for PDAC were able to follow a physical training sequence during each period of medical treatment. However, no re-nutrition program was associated, and the effect on OS was not studied. Weight stabilization has been proven to improve overall survival and quality of life in patients with unresectable pancreatic cancer [25], but no similar study has been conducted on BRPC or LAPC. Recent position paper [10] of the International Study Group of Pancreatic Surgery highlighted the need for considering preoperative nutritional support in order to decrease postoperative complications in patients meeting 1 out of the 4 following criteria: WL *>* 15% within 6 months, BMI *<* 18.5 kg/m^2^, subjective global assessment grade C or nutritional risk score *>* 5, and serum albumin level *<* 30 g/L. Here, we at the same time extend the concerned population to all LAPC and BRPC patients (before decision of surgery) and simplify the criteria to a WL > 5% and PS > 0. Setting up a multimodal prehabilitation program of renutrition and physical activity based on WL and PS at diagnosis, to improve PS and reverse the weight curve during an induction treatment, could thus be beneficial not only to LAPC and BRPC patients but also to the minority of up-front resectable patients if the recent trend of systematic induction treatment is confirmed and commonly approved. A program based on physical exercise and nutrition care could also be beneficial to the obese population, as our results revealed, consistently with the literature [26], that OS in obese patients of the resected group was worse.

### Limitations

Our study has limitations: firstly, in our 454-patient cohort, the induction treatment regimen was heterogeneous, and we could not evaluate the effect of this heterogeneity on our results. Therefore, there is no clear recommendation on the optimal induction sequence, and either chemotherapy alone or chemoradiation had been administered, according to the period of inclusion which included different induction regimen at our institution. We did not focus on these differences in the induction regimen, but it would be interesting to study it separately. Secondly, this clinical study does not take into account tumor biology. It is now known that biological heterogeneity underlies PDAC development, treatment resistance, and prognosis [27]. If the epigenetic profile of a tumor seems to explain its aggressiveness, none has yet been correlated to the clinical impact for the patient. Cachexia in PDAC has been explored in fundamental studies, and mechanisms are not completely elucidated, even if some tracks are emerging, such as cytokine profiling in tumoral tissue [28]. However, the relationships between metabolism, WL, and OS in PDAC are not established [8]. Unfortunately, neither biological nor clinical factor of poor prognosis through WL or poor PS has yet been described. The origin of WL and poor PS are not even known: are they a clinical consequence of the disease or do they reflect its aggressiveness?

## Conclusions and perspectives

Our results propose that continuing losing some weight during the induction treatment and bearing a poor PS are independent factors of poor prognosis in BRPC and LAPC patients, but they are dynamic criteria that have to be monitored for a few weeks. Here, we chose to look at the variation of these clinical items at reevaluation staging (after a 3-month induction treatment), but an earlier reevaluation could also be significant in order to initiate a “reconditioning” program as close to the diagnosis as possible. Yet, studies are being conducted (9 identified ongoing studies in ClinicalTrials.gov) to assess the effect of a pre-habilitation program in pancreatic cancer surgery, but these studies start when the surgery has already been planned. Thus, they do not include patients at diagnosis, whose therapeutic sequence may change due to physical and nutritional protocols. Will re-nutrition and physical rehabilitation be efficient to rectify the prognosis of the patients, or should these patients be classified in a palliative category as soon as they present a CWL and a PS > 1? To date, the answer to this question remains unknown, but recent international consensus include a PS > 2 as a clinical criteria of BRPC [29]. It is a fact that in daily clinical practice, physicians lack reconditioning propositions towards these frail patients. Clinical effects on the OS of a program aimed at improving PS and reverse weighing curve in patients with BRPC or LAPC should be assessed in a prospective study.

## Data Availability

All the data are available in the clinical database “CHIRPAN,” which has previously been approved by the French “Commission Nationale Informatique et Liberté.”
